# Tumour mutation burden drives survival outcomes in pancreatic ductal adenocarcinoma and enables noninvasive prediction via dual-layer spectral CT

**DOI:** 10.1186/s13244-026-02216-5

**Published:** 2026-02-09

**Authors:** Jiawei Liu, Siya Shi, Meicheng Chen, Jiadan Luo, Luyong Wei, Mingjie Chen, Zujiang Shi, Liqin Wang, Yanji Luo, Shi-Ting Feng

**Affiliations:** Department of Radiology, The First Affiliated Hospital, Sun Yat-sen University, Guangzhou, China

**Keywords:** Pancreatic ductal adenocarcinoma, Tumour mutation burden, Prognosis, Dual-layer spectral CT, Immunotherapy

## Abstract

**Objectives:**

To evaluate the prognostic significance of tumour mutation burden (TMB) in pancreatic ductal adenocarcinoma (PDAC) and explore the performance of dual-layer spectral CT (DLCT) for noninvasive TMB evaluation.

**Materials and methods:**

This retrospective analysis enroled patients with histopathologically confirmed PDAC who underwent DLCT between June 2019 and December 2023. Clinical, qualitative radiological, and quantitative conventional CT and DLCT parameters were evaluated. Survival analysis evaluated TMB’s association with progression-free survival (PFS) and identified an optimal TMB cutoff. Independent TMB predictors were identified through univariable and LASSO regression. Predictive performance was quantified via receiver operating characteristic and precision-recall curve assessments.

**Results:**

Among 75 patients (mean age 60.4 ± 11.2 years; 41 males, 34 females), median TMB was 2.13 mut/Mb (interquartile range: 1.00–4.26). A 5 mut/Mb cutoff revealed distinct prognostic groups, with high-TMB cases exhibiting better PFS (median PFS: 7 vs 5 months, *p* = 0.02). Normalised iodine concentration in the pancreatic phase (nICa) was the sole independent TMB predictor (area under the curve [AUC] = 0.901; cutoff = 0.089; accuracy = 89.3% [89.1–89.6%], sensitivity = 81.8% [59.0–100%], specificity = 90.6% [83.5–97.8%]), surpassing conventional CT attenuation metrics (nCTa, AUC = 0.834), peripancreatic tumour infiltration (AUC = 0.679), and their combined model (AUC = 0.864) with significant net reclassification improvement (all *p* < 0.05). Precision-recall curve validation reinforced nICa’s superior predictive capacity. Patients classified by nICa-predicted high TMB status demonstrated better PFS (median PFS: 7 vs 5 months, *p* = 0.04).

**Conclusion:**

Elevated TMB is a positive biomarker for PFS in PDAC. DLCT-derived nICa facilitates precise, noninvasive TMB prediction, outperforming conventional imaging parameters and supporting its potential role in therapeutic stratification.

**Critical relevance statement:**

Elevated tumour mutational burden (TMB) in PDAC correlated with prolonged PFS. DLCT provided noninvasive, accurate TMB quantification, enabling meaningful survival stratification.

**Key Points:**

- High TMB in patients with PDAC portends better PFS, particularly those receiving combination immunotherapy.
- A clinically applicable TMB cutoff of 5 mut/Mb was identified, stratifying patients into biologically distinct low- and high-TMB prognostic groups.
- DLCT-derived pancreatic phase normalized iodine concentration emerged as a superior noninvasive TMB biomarker compared to conventional imaging parameters.

**Graphical Abstract:**

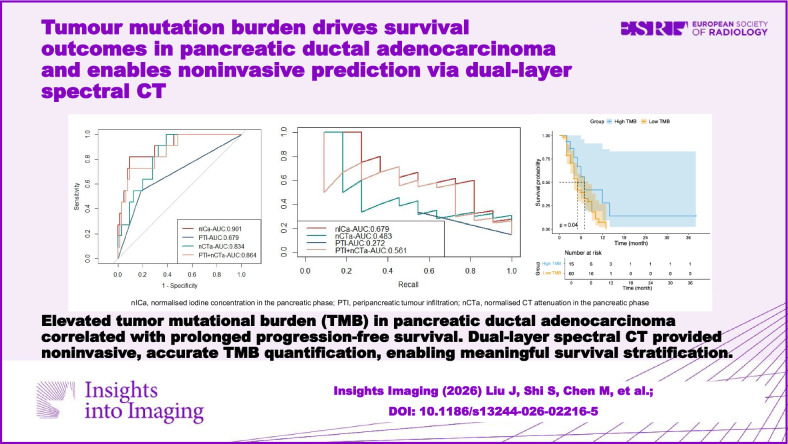

## Introduction

While chemotherapy remains the cornerstone of treatment for advanced pancreatic ductal adenocarcinoma (PDAC), its therapeutic efficacy remains modest, with median survival improvements measured in mere months [1, 2]. The advent of immunotherapy has introduced promising therapeutic avenues, with current National Comprehensive Cancer Network (NCCN) guidelines advocating for immune biomarker profiling in locally advanced or metastatic PDAC cases, coupled with strategic deployment of immunotherapeutic agents in conjunction with chemotherapeutic regimens where clinically indicated [3, 4]. However, significant interpatient heterogeneity in therapeutic response poses a formidable challenge, as evidenced by divergent clinical trial outcomes—while some studies failed to demonstrate survival benefits in unselected cohorts [5], others reported enhanced resectability rates and prolonged overall survival [6]. This therapeutic conundrum underscores the pressing need for robust predictive biomarkers to guide precision medicine approaches in PDAC immunotherapy.

Tumour mutation burden (TMB), operationally defined as the somatic coding mutation density per megabase, provides a quantitative metric of genomic instability within malignant cells [7]. The fundamental immunological premise suggests that elevated TMB correlates with increased neoantigen diversification, thereby potentiating immune surveillance and anti-tumour cytotoxicity [8]. While TMB has emerged as a validated predictive biomarker across multiple solid malignancies [9–11], its prognostic significance in PDAC, particularly in the context of combination immunotherapy, remains inadequately characterized. Besides, current TMB quantification methodologies primarily employ next-generation sequencing (NGS) of tumour specimens—an approach encumbered by substantial financial burden, technical complexity, and procedural invasiveness [12, 13]. These limitations highlight the unmet clinical need for economical, noninvasive, and readily accessible alternatives for TMB assessment.

Dual-layer spectral CT (DLCT) represents a technological advancement over conventional CT, employing simultaneous dual-energy photon detection through stratified detector arrays [14]. This novel imaging modality yields quantitative parametric data beyond conventional CT capabilities, facilitating comprehensive characterization of tumoural microenvironments and predictive modelling of therapeutic responses in PDAC [15, 16]. However, the potential utility of DLCT-derived parameters for predicting TMB status in PDAC patients remains entirely unexplored in the current scientific literature.

Thus, the present investigation aims to evaluate the prognostic significance of TMB in PDAC and the predictive capabilities of DLCT for noninvasive TMB assessment.

## Materials and methods

### Patient

**Fig. 1:**
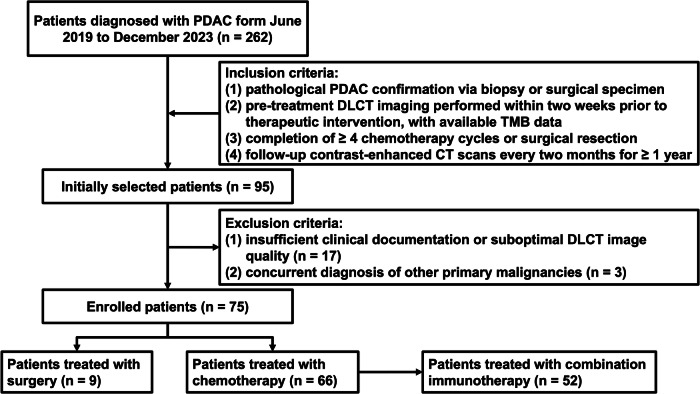
Flowchart of the study enrolment procedure. PDAC, pancreatic ductal adenocarcinoma; DLCT, dual-layer spectral CT; TMB, tumour mutation burden

This retrospective study, approved by our Institutional Review Board (The First Affiliated Hospital of Sun Yat-sen University institution approved this single-centre study (approval number: 2021 [721])), with waiver of informed consent, analysed consecutive patients with histopathologically confirmed PDAC who underwent contrast-enhanced abdominal DLCT within 14 days before treatment initiation between June 2019 and December 2023. Inclusion criteria required: (1) pathological PDAC confirmation via biopsy or surgical specimen, (2) pre-treatment DLCT imaging performed within two weeks prior to therapeutic intervention, with available TMB data, (3) completion of ≥ 4 chemotherapy cycles or surgical resection, and (4) follow-up contrast-enhanced CT scans every two months for ≥ 1 year. Key exclusion criteria encompassed: (1) insufficient clinical documentation or suboptimal DLCT image quality, and (2) concurrent diagnosis of other primary malignancies. The patient selection process is detailed in Fig. 1.

### DLCT image acquisition and image-processing

All study participants were examined using a DLCT scanner (IQon Spectral CT; Philips Healthcare) operated at 120 kVp tube voltage with automated tube current modulation. The acquisition protocol was standardized with the following parameters: gantry rotation time of 0.5 s, helical pitch factor of 0.798, 512 × 512 reconstruction matrix, 64 × 0.625 mm collimation width, 1 mm slice thickness, and 0.8 mm reconstruction increment. Participants maintained a fasting state for at least 4 h preceding the examination. An intravenous bolus injection of non-ionic iodinated contrast medium (iopromide, Ultravist 370; Bayer Schering Pharma) was administered at 3 mL/s (1.5 mL/kg body weight, not exceeding 100 mL total volume), followed by a 40 mL saline chaser. Biphasic imaging was performed with pancreatic phase acquisition at 35 s and portal venous phase acquisition at 65 s post-injection.

All spectral datasets were reconstructed and archived on a dedicated advanced visualization platform (IntelliSpace Portal 10.0, Philips Healthcare). Conventional polyenergetic images were reconstructed at 120 kVp using hybrid iterative reconstruction (iDose4, level 3; Philips Healthcare). Spectral decomposition algorithms were employed to generate: (a) virtual monoenergetic images (VMIs) at 40, 70, and 100 keV, (b) quantitative iodine concentration (IC) maps, and (c) effective atomic number (Zeff) parametric images.

### Image analysis

Two fellowship-trained abdominal radiologists (with respective 5 and 10 years of subspecialty experience in pancreatic imaging) conducted blinded, independent evaluations of all imaging studies. Both interpreters remained completely masked to all clinical and pathological data. Qualitative radiological characteristics were determined through consensus evaluation, while quantitative measurements were performed separately by each radiologist. To resolve any interpretive discrepancies, a senior radiologist with 22 years of pancreatic imaging expertise served as an independent arbiter to establish definitive radiological findings.

The qualitative radiological evaluation encompassed comprehensive assessment of multiple tumour characteristics, including tumour localization (head/uncinate vs body/tail), tumour size, necrotic components, peripancreatic tumour infiltration (PTI), alterations in ductal architecture (pancreaticobiliary systems), parenchymal atrophy, and critical spatial relationships with major vascular structures (coeliac trunk artery, common hepatic artery, superior mesenteric artery, superior mesenteric vein, and portal vein). Tumour size was precisely quantified through axial measurement of the maximum perpendicular diameter [17]. Necrotic regions were radiologically defined as hypodense areas demonstrating < 20 Hounsfield units (HU) on noncontrast images, with absent significant enhancement (< 10 HU increase) during both pancreatic and portal venous phases [14]. PTI manifested as neoplastic extension beyond the pancreatic capsule into adjacent adipose tissue, characterized by nodular, linear, or irregular enhancement patterns on contrast-enhanced imaging [17]. Ductal abnormalities were objectively defined as main pancreatic duct dilatation (> 3 mm diameter) [18] or biliary tree dilation (> 2 mm hepatic duct or > 8 mm common bile duct diameters) [19]. Parenchymal atrophy was quantitatively diagnosed when the pancreatic width measured < 10 mm across ≥ 50% of the glandular length [20]. Vascular interfaces were systematically classified using angular quantification: coeliac, hepatic, and superior mesenteric arterial relationships were stratified as no contact, ≤ 180° circumference involvement, or > 180° encasement [17]. Venous (superior mesenteric/portal) tumour relationships were categorized into a three-tier system: (1) no contact or ≤ 180° abutment with preserved venous contour; (2) > 180° encirclement or ≤ 180° contact with contour deformity/thrombosis, yet maintaining adequate proximal/distal segments permitting oncologically safe resection with venous reconstruction; and (3) unresectable status due to complete venous occlusion precluding reconstruction [17].

All quantitative measurements were independently performed by two radiologists utilizing multiple imaging datasets, including conventional 120 kVp polyenergetic images, VMIs reconstructed at 40, 70, and 100 keV, IC maps, and Zeff images. To evaluate measurement reproducibility, the more experienced fellowship-trained abdominal radiologist (with 10 years of subspecialty experience in pancreatic imaging) repeated all quantitative assessments following a one-month washout period. For each tumour, standardized region-of-interest (ROI) placements were performed at the level of maximal axial diameter using 40 keV VMI datasets [21], with strict adherence to a minimum area threshold of 100 mm². Rigorous exclusion criteria were applied to avoid confounding measurements, with particular attention paid to omitting calcified regions, vascular structures, and necrotic components. These precisely delineated ROIs were then systematically propagated to all corresponding imaging datasets (conventional 120 kVp images, 70/100 keV VMIs, IC maps, and Zeff images) for comprehensive quantification of CT attenuation values, ICs, and Zeff. Concurrently, standardized aortic reference measurements were obtained at identical axial levels, with careful avoidance of atherosclerotic plaque-bearing segments while maintaining the same minimum 100 mm² area requirement. All quantitative parameters were recorded as mean values derived from both radiologists’ measurements to enhance data reliability. A standardized nomenclature was employed, where the prefix “*n*” denotes normalised values, while the suffixes “*a*” and “*v*” differentiate pancreatic-phase and portal venous-phase parameters, respectively. The spectral attenuation curve slope (K) mathematically derived from VMI datasets, and the portal venous-to-pancreatic phase relative attenuation ratio were calculated according to the following equations [22]:

$${nICa}=\frac{{{ICa}}_{{tumour}}}{{{ICa}}_{{aorta}}}$$

$$K70/40a=\frac{n40a-n70a}{70-40}$$

$${IC}\, {ratio}=\frac{{nICv}}{{nICa}}$$

### Clinical and pathological characteristics

A comprehensive dataset of clinical variables was meticulously accrued by an independent radiologist, who was not participating in imaging analyses to eliminate potential assessment bias, through a systematic review of the electronic patient files. This dataset included therapeutic strategies, demographic parameters (age, sex, body mass index [BMI]), clinical manifestations (jaundice status, abdominal pain), comorbid conditions (diabetes mellitus, smoking history), serum biomarker levels (carbohydrate antigen 19-9), and survival outcomes (progression-free survival [PFS]).

TMB quantification employed whole-exome sequencing analysis of DNA extracted from formalin-fixed, paraffin-embedded tumour samples procured either through biopsy or surgical resection [23]. Library preparation utilized a targeted probe capture methodology, enabling high-coverage next-generation sequencing of the neoplastic tissue. The genomic analysis encompassed mutational profiling across 575 oncologically relevant genes, representing approximately 2.2 million bases of coding sequence in the human genome. All sequencing data underwent stringent quality control measures, with final analyses restricted to samples achieving a minimum mean sequencing depth of 200×. The resulting mutational load was expressed as the count of validated somatic, non-synonymous mutations per megabase of sequenced DNA.

### Statistical analysis

Statistical analyses were performed utilizing three distinct software platforms: SPSS version 25.0 (SPSS Inc., Chicago, IL, USA), MedCalc version 20.218 (MedCalc Software Ltd.), and R (R Development Core Team). The putative association between TMB and PFS was evaluated through Cox proportional hazards regression modelling, with optimal TMB threshold determination established via receiver operating characteristic (ROC) curve analysis. To enhance clinical applicability, we subsequently employed an integer-based TMB cutoff to stratify patients into high- and low-TMB cohorts, followed by survival analysis using the Kaplan–Meier method. Interobserver concordance for continuous variables was quantified through calculation of intraclass correlation coefficients (ICCs), interpreted according to conventional benchmarks: 0–0.20 (slight), 0.21–0.40 (fair), 0.41–0.60 (moderate), 0.61–0.80 (substantial), and 0.81–1.00 (near-perfect agreement). Continuous data were expressed as mean ± standard deviation for normally distributed parameters or median (interquartile range) for non-normally distributed variables, with comparative analysis conducted using either Student’s *t*-test or Mann–Whitney *U*-test, as dictated by data distribution. Categorical variables were presented as frequencies (percentages) and analysed via chi-square test or Fisher’s exact test. Univariable logistic regression analysis was performed to preliminarily identify TMB-associated variables. Variables found to be statistically significant in the univariable model were subsequently included in a least absolute shrinkage and selection operator (LASSO) regression, aimed at refining the predictive model for TMB assessment and enhancing both its accuracy and interpretability. Variables identified through the LASSO procedure were then entered into a multivariable logistic regression analysis to ascertain independent predictors. Model performance was comprehensively evaluated through ROC curve analysis supplemented by net reclassification improvement (NRI) and integrated discrimination improvement (IDI) metrics. Given the inherent class imbalance between TMB groups, we implemented the F_1_ score and precision-recall (PR) curve analysis—measures that provide more robust performance evaluation for imbalanced datasets [24]. Finally, we generated PFS Kaplan–Meier curves stratified by model-predicted TMB classifications, with between-group comparisons performed via log-rank test. A *p*-value of < 0.05 was considered statistically significant.

## Results

### Prognostic values of TMB for PDAC

**Fig. 2:**
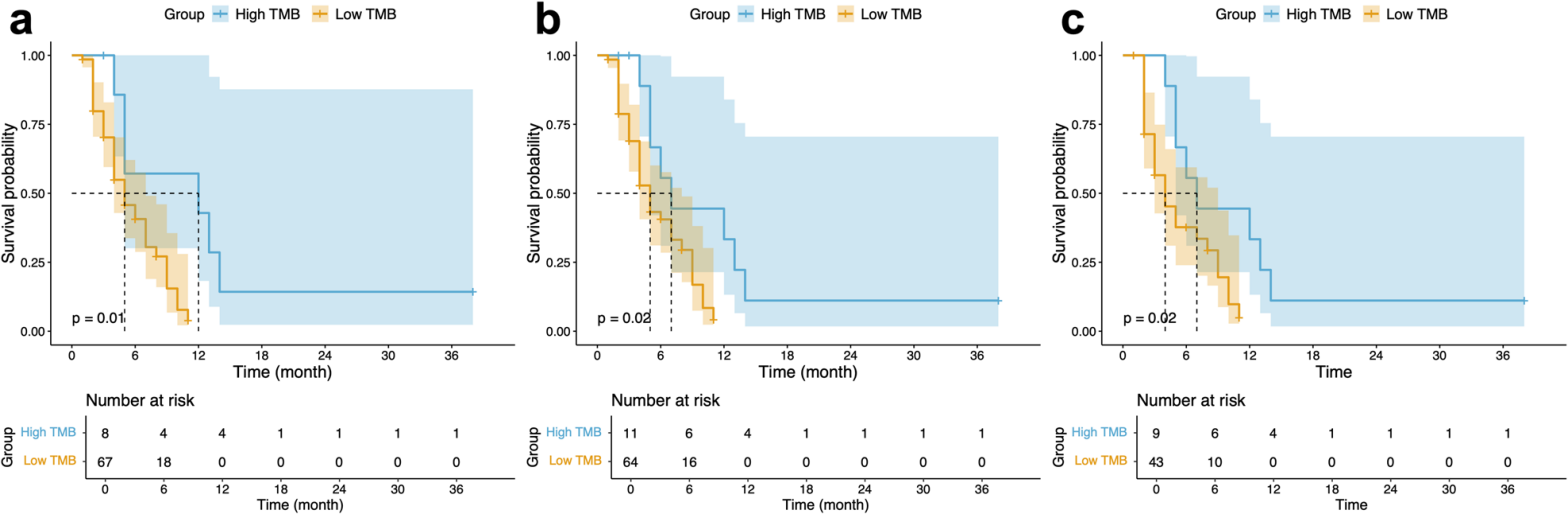
Kaplan–Meier survival curves on the high and low TMB groups. **a** Comparison of progression-free survival between high and low TMB groups in all patients, stratified using the optimal cutoff value of 5.03. **b** Comparison of progress-free survival between high and low TMB groups in all patients, stratified using the integrated cutoff value of 5. **c** Comparison of progress-free survival between high and low TMB groups in 52 patients receiving combination immunotherapy, stratified using the integrated cutoff value of 5. TMB, tumour mutation burden

A total of 75 patients (mean age, 60.40 ± 11.24 years; 41 males, 34 females) were finally enroled. The median TMB was 2.13 mut/Mb (interquartile range: 1.00–4.26). The relationship between TMB and PFS was analysed using ROC analysis, which determined an optimal cutoff value of 5.03 mut/Mb. Patients with TMB greater than 5.03 mut/Mb were classified into the high TMB group (8 patients), and those with TMB ≤ 5.03 mut/Mb were classified into the low TMB group (67 patients). Kaplan–Meier survival curves are shown in Fig. 2a. The results indicated that PFS was superior in the high TMB group compared to the low TMB group (median PFS: 12 vs 5 months, *p* = 0.01).

For clinical convenience, we used an integer cutoff of 5 mut/Mb, classifying PDAC patients with TMB ≥ 5 mut/Mb as the high TMB group (11 patients) and those with TMB < 5 mut/Mb as the low TMB group (64 patients). Kaplan–Meier survival curves are shown in Fig. 2b. The results further indicated that PFS was significantly better in the high TMB group compared to the low TMB group (median PFS: 7 vs 5 months, *p* = 0.02), with a hazard ratio of 0.89 (95% confidence interval [CI]: 0.80–0.99, *p* = 0.04). Using the same threshold of 5 mut/Mb, survival analysis was conducted on 52 PDAC patients receiving combination immunotherapy. Kaplan–Meier survival curves are shown in Fig. 2c. The results also indicated that PFS was significantly better in the high TMB group (9 patients) compared to the low TMB group (43 patients) (median PFS: 7 vs 4 months, *p* = 0.02), with a hazard ratio of 0.88 (95% CI: 0.78–0.99, *p* = 0.045).

### Relevant clinical and qualitative radiological characteristics for predicting TMB

The clinical and qualitative radiological characteristics of both groups are summarized in Table 1. Only PTI demonstrated a statistically significant difference between the high and low TMB groups. There were no statistically significant differences between the groups for age, sex, BMI, smoking history, diabetes, jaundice, abdominal pain, CA 19-9 level, tumour location, tumour size, tumour necrosis, main pancreatic duct dilation, bile duct dilation, pancreatic atrophy, and arterial or venous contact (all *p* ≥ 0.05).

**Table 1:** The differences of clinical and qualitative radiological characteristics between the low and high TMB groups

Variables	Total (*n* = 75)	Low group (*n* = 64)	High group (*n* = 11)	*p* value^a^
Age	60.40 ± 11.24	60.31 ± 9.89	60.91 ± 17.84	0.87
Sex				0.75
Female	34 (45)	30 (47)	4 (36)	
Male	41 (55)	34 (53)	7 (64)	
BMI	21.88 (20.16, 23.70)	21.92 (20.31, 23.68)	21.19 (18.04, 24.10)	0.44
Smoking				0.72
No	56 (75)	47 (73)	9 (82)	
Yes	19 (25)	17 (27)	2 (18)	
Diabetes				> 0.999
No	61 (81)	52 (81)	9 (82)	
Yes	14 (19)	12 (19)	2 (18)	
Jaundice				0.44
No	59 (79)	49 (77)	10 (91)	
Yes	16 (21)	15 (23)	1 (9)	
Abdominal pain				0.06
No	20 (27)	14 (22)	6 (55)	
Yes	55 (73)	50 (78)	5 (45)	
CA 19-9 (U/mL)	481 (38, 2732)	413 (28, 2666)	1176 (91, 2429)	0.65
Tumour location				0.25
Body/tail	36 (48)	33 (52)	3 (27)	
Head/uncinate	39 (52)	31 (48)	8 (73)	
Tumour diameter (mm)	42.0 (29.0, 50.5)	40.0 (28.0, 50.3)	43.0 (38.0, 49.0)	0.43
Necrosis				> 0.999
No	31 (41)	27 (42)	4 (36)	
Yes	44 (59)	37 (58)	7 (64)	
PTI				0.02^*^
No	18 (24)	12 (19)	6 (55)	
Yes	57 (76)	52 (81)	5 (45)	
Main pancreatic duct dilation				0.75
No	34 (45)	30 (47)	4 (36)	
Yes	41 (55)	34 (53)	7 (64)	
Bile duct dilation				0.74
No	51 (68)	44 (69)	7 (64)	
Yes	24 (32)	20 (31)	4 (36)	
Pancreatic atrophy				0.72
No	57 (76)	49 (77)	8 (73)	
Yes	18 (24)	15 (23)	3 (27)	
Contact with the coeliac trunk artery				0.17
No contact	57 (76)	46 (72)	11 (100)	
Contact of ≤ 180°	7 (9)	7 (11)	0 (0)	
Contact of > 180°	11 (15)	11 (17)	0 (0)	
Contact with the common hepatic artery				0.87
No contact	53 (71)	44 (69)	9 (82)	
Contact of ≤ 180°	8 (11)	7 (11)	1 (9)	
Contact of > 180°	14 (19)	13 (20)	1 (9)	
Contact with the superior mesenteric artery				0.56
No contact	55 (73)	45 (70)	10 (91)	
Contact of ≤ 180°	10 (13)	9 (14)	1 (9)	
Contact of > 180°	10 (13)	10 (16)	0 (0)	
Contact with the superior mesenteric vein or the portal vein				0.75
Regular vein contour	33 (44)	27 (42)	6 (55)	
Reconstructible	30 (40)	26 (41)	4 (36)	
Unreconstructible	12 (16)	11 (17)	1 (9)	

*TMB* tumour mutation burden, *BMI* body mass index, *CA 19-9* carbohydrate antigen 19-9, *PTI* peripancreatic tumour infiltration

^a^ Continuous variables were reported as mean ± standard deviation or median (interquartile range) and compared using either the *t*-test or the Mann–Whitney *U*-test. Categorical variables were summarized as frequencies (percentages) and analysed using the chi-square test or Fisher’s exact test

^*^ The parameter is statistically significant (*p* < 0.05) and is shown in bold type

### Relevant quantitative parameters for predicting TMB

The ICCs between the measurements of the two radiologists for all quantitative parameters ranged from 0.73 to 0.96, indicating substantial to near-perfect agreement. The ICCs for repeated measurements by the same radiologist ranged from 0.81 to 0.99, demonstrating near-perfect intra-observer agreement.

Among the conventional CT and DLCT quantitative parameters, nCTa, n40a, n70a, n100a, nICa, and nZeffa, which were obtained in the pancreatic phase, exhibited significantly elevated values in the high TMB group compared to the low TMB group (*p* < 0.05). Additionally, several parameters obtained in the portal venous phase, including nCTv, n40v, n70v, and nICv, were also higher in the high TMB group than the low TMB group (*p* < 0.05). Only the IC ratio demonstrated a statistically significant difference for the portal venous-to-pancreatic phase relative attenuation ratio. No statistically significant differences were observed for the remaining parameters (summarized in Table 2).

**Table 2:** The differences in quantitative radiological characteristics between the low and high TMB groups

Variables	Total (*n* = 75)	Low group (*n* = 64)	High group (*n* = 11)	*p* value^a^
nCTa	0.19 ± 0.04	0.18 ± 0.04	0.23 ± 0.03	< 0.001^*^
nCTv	0.36 (0.32, 0.41)	0.35 (0.32, 0.40)	0.41 (0.40, 0.44)	0.005^*^
CT ratio	1.95 (1.72, 2.29)	1.98 (1.72, 2.34)	1.82 (1.75, 2.02)	0.30
n40a	0.10 (0.09, 0.12)	0.10 (0.08, 0.11)	0.13 (0.12, 0.17)	< 0.001^*^
n70a	0.18 ± 0.04	0.17 ± 0.04	0.22 ± 0.04	< 0.001^*^
n100a	0.28 ± 0.06	0.27 ± 0.05	0.33 ± 0.07	0.003^*^
nICa	0.07 (0.05, 0.08)	0.06 (0.04, 0.08)	0.11 (0.09, 0.14)	< 0.001^*^
nZeffa	0.70 ± 0.03	0.69 ± 0.03	0.72 ± 0.02	0.002^*^
n40v	0.26 (0.21, 0.29)	0.25 (0.21, 0.28)	0.30 (0.28, 0.33)	< 0.001^*^
n70v	0.37 (0.32, 0.40)	0.35 (0.32, 0.39)	0.40 (0.39, 0.42)	0.001^*^
n100v	0.48 (0.41, 0.52)	0.47 (0.41, 0.52)	0.50 (0.48, 0.51)	0.20
nICv	0.18 (0.15, 0.23)	0.18 (0.15, 0.22)	0.23 (0.21, 0.28)	0.001^*^
nZeffv	0.82 (0.81, 0.84)	0.82 (0.81, 0.83)	0.83 (0.82, 0.85)	0.05
K70/40a	0.24 (0.20, 0.29)	0.24 (0.20, 0.29)	0.25 (0.21, 0.26)	0.91
K100/70a	0.33 (0.27, 0.38)	0.33 (0.27, 0.38)	0.33 (0.29, 0.35)	0.96
K100/40a	0.28 (0.24, 0.33)	0.28 (0.23, 0.34)	0.29 (0.25, 0.31)	0.95
K70/40 v	0.36 (0.28, 0.42)	0.37 (0.28, 0.43)	0.35 (0.28, 0.36)	0.41
K100/70v	0.40 (0.28, 0.46)	0.41 (0.29, 0.48)	0.37 (0.24, 0.40)	0.20
K100/40v	0.38 (0.29, 0.44)	0.38 (0.29, 0.45)	0.36 (0.24, 0.38)	0.24
40 keV ratio	2.44 (2.00, 2.87)	2.44 (2.06, 2.92)	2.46 (1.96, 2.64)	0.29
70 keV ratio	2.06 ± 0.51	2.09 ± 0.53	1.89 ± 0.31	0.25
100 keV ratio	1.68 (1.50, 1.89)	1.76 (1.52, 1.94)	1.56 (1.40, 1.68)	0.08
IC ratio	2.72 (2.20, 3.69)	2.77 (2.27, 3.79)	2.50 (1.83, 2.79)	0.04^*^
Zeff ratio	1.18 (1.15, 1.22)	1.18 (1.15, 1.22)	1.17 (1.15, 1.19)	0.45

*TMB* tumour mutation burden, *40/70/100* virtual monoenergetic images at 40/70/100 keV, *IC* iodine concentration, *Zeff* effective atomic number

The prefix “*n*” was used to indicate normalisation. The suffixes “*a*” and “*v*” were appended to the indicators to denote pancreatic and portal venous phase parameters, respectively. The prefix “K” was used to represent the slope of the spectral attenuation curve. The “ratio” was used to refer to the portal venous-to-pancreatic phase relative attenuation ratio

^a^ Continuous variables were reported as mean ± standard deviation or median (interquartile range) and compared using either the *t*-test or the Mann–Whitney *U*-test

^*^ The parameter is statistically significant (*p* < 0.05) and is shown in bold type

### Predictive performance of factors for predicting TMB

In univariable analysis, abdominal pain (odds ratio [OR] = 0.23, 95% CI: 0.06–0.88, *p* = 0.03), PTI (OR = 0.19, 95% CI: 0.05–0.74, *p* = 0.02), nCTa (OR = 1.42, 95% CI: 1.14–1.76, *p* = 0.001), and nCTv (OR = 1.11, 95% CI: 1.01–1.23, *p* = 0.03) were identified as significant risk factors for TMB based on clinical and conventional CT characteristics (Table 3). In the subsequent multivariable analysis using forward selection, only PTI (adjusted OR = 0.22, 95% CI: 0.05–0.88, *p* = 0.02) and nCTa (adjusted OR = 1.40, 95% CI: 1.12–1.74, *p* = 0.001) remained as independent predictors of TMB.

**Table 3:** The univariable analysis of clinical, conventional CT, and DLCT parameters

Clinical and conventional CT characteristics				DLCT parameters			
Variables	OR	95% CI	*p* values	Variables	OR	95% CI	*p* values
Age	1.01	0.95–1.06	0.87	n40a	1.60	1.22–2.09	0.001^*^
Sex	1.54	0.41–5.80	0.52	n70a	1.39	1.14-1.70	0.001^*^
BMI	1.07	0.94–1.21	0.29	n100a	1.20	1.04–1.37	0.01^*^
Smoking	0.61	0.12–3.13	0.56	nICa	1.69	1.28–2.22	< 0.001^*^
Diabetes	0.96	0.18–5.04	0.96	nZeffa	1.50	1.13–2.00	0.006^*^
Jaundice	0.33	0.04–2.76	0.30	n40v	1.17	1.03–1.32	0.01^*^
Abdominal pain	0.23	0.06–0.88	0.03^*^	n70v	1.13	1.01–1.25	0.03^*^
CA 19-9	1.00	1.00–1.00	0.32	n100v	1.04	0.97–1.11	0.27
Tumour location	2.84	0.69–11.68	0.15	nICv	1.14	1.03–1.26	0.01^*^
Tumour diameter	1.01	0.97–1.04	0.70	nZeffv	1.01	0.97–1.06	0.63
Necrosis	1.28	0.34–4.80	0.72	K70/40a	1.03	0.96–1.10	0.47
PTI	0.19	0.05–0.74	0.02^*^	K100/70a	1.03	0.97–1.10	0.36
Main pancreatic duct dilation	1.54	0.41–5.80	0.52	K100/40a	1.04	0.96–1.11	0.37
Bile duct dilation	1.26	0.33–4.79	0.74	K70/40 v	0.99	0.95–1.04	0.70
Pancreatic atrophy	1.23	0.29–5.21	0.78	K100/70v	0.98	0.95–1.01	0.22
Contact with the coeliac trunk artery	0	0–Inf	0.99	K100/40v	0.98	0.94–1.02	0.32
Contact with the common hepatic artery	0.63	0.23–1.70	0.36	40 keV ratio	0.99	0.98–1.00	0.22
Contact with the superior mesenteric artery	0.29	0.05–1.71	0.17	70 keV ratio	0.99	0.98–1.01	0.24
Contact with the superior mesenteric vein or the portal vein	0.66	0.25–1.71	0.39	100 keV ratio	0.99	0.97–1.00	0.15
nCTa	1.42	1.14–1.76	0.001^*^	IC ratio	0.99	0.98–1.00	0.052
nCTv	1.11	1.01–1.23	0.03^*^	Zeff ratio	0.99	0.92–1.06	0.71
CT ratio	0.99	0.98–1.01	0.31				

The prefix “*n*” was used to indicate normalisation. The suffixes “*a*” and “*v*” were appended to the indicators to denote pancreatic and portal venous phase parameters, respectively. The prefix “K” was used to represent the slope of the spectral attenuation curve. The “ratio” was used to refer to the portal venous-to-pancreatic phase relative attenuation ratio

*DLCT* dual-layer spectral CT, *OR* odds ratio, *CI* confidence interval, *BMI* body mass index, *CA 19-9* carbohydrate antigen 19-9, *PTI* peripancreatic tumour infiltration, *40/70/100* VMI at 40/70/100 keV, *IC* iodine concentration, *Zeff* effective atomic number

^*^ The parameter is statistically significant (*p* < 0.05) and is shown in bold type

**Fig. 3:**
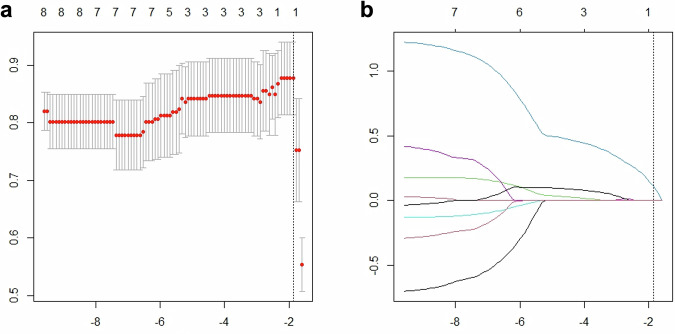
Feature selection of DLCT parameters for LASSO regression. **a** represents the selection of the tuning parameter (λ) for LASSO regression. The area under the curve (AUC) was plotted against log(λ), and five-fold cross-validation was used to select the optimal value of λ. **b** shows the coefficient distribution of quantitative parameters in DLCT. A vertical dashed line is plotted at the selected λ, where nICa was identified as the only non-zero coefficient. DLCT, dual-layer spectral CT; LASSO, least absolute shrinkage and selection operator; nICa, normalised iodine concentration in the pancreatic phase

In the univariable analysis of DLCT parameters, n40a (OR = 1.60, 95% CI: 1.22–2.09, *p* = 0.001), n70a (OR = 1.39, 95% CI: 1.14–1.70, *p* = 0.001), n100a (OR = 1.20, 95% CI: 1.04–1.37, *p* = 0.01), nICa (OR = 1.69, 95% CI: 1.28–2.22, *p* < 0.001), nZeffa (OR = 1.50, 95% CI: 1.13–2.00, *p* = 0.006), n40v (OR = 1.17, 95% CI: 1.03–1.32, *p* = 0.01), n70v (OR = 1.13, 95% CI: 1.01–1.25, *p* = 0.03) and nICv (OR = 1.14, 95% CI: 1.03–1.26, *p* = 0.01) were identified as significant risk factors for TMB (Table 3). The IC ratio, despite showing a trend toward a difference in distribution, did not reach statistical significance in univariable analysis (*p* = 0.052). Among these parameters, only nICa was retained after LASSO regression (Fig. 3).

Multivariable analysis showed that nICa (adjusted OR = 1.53, 95% CI: 1.10–2.14, *p* = 0.01) derived from DLCT parameters was the only independent predictors of TMB, in contrast to PTI (adjusted OR = 0.26, 95% CI: 0.05–1.46, *p* = 0.13) and nCTa (adjusted OR = 1.13, 95% CI: 0.87–1.47, *p* = 0.35) from clinical and conventional CT characteristics.

**Fig. 4:**
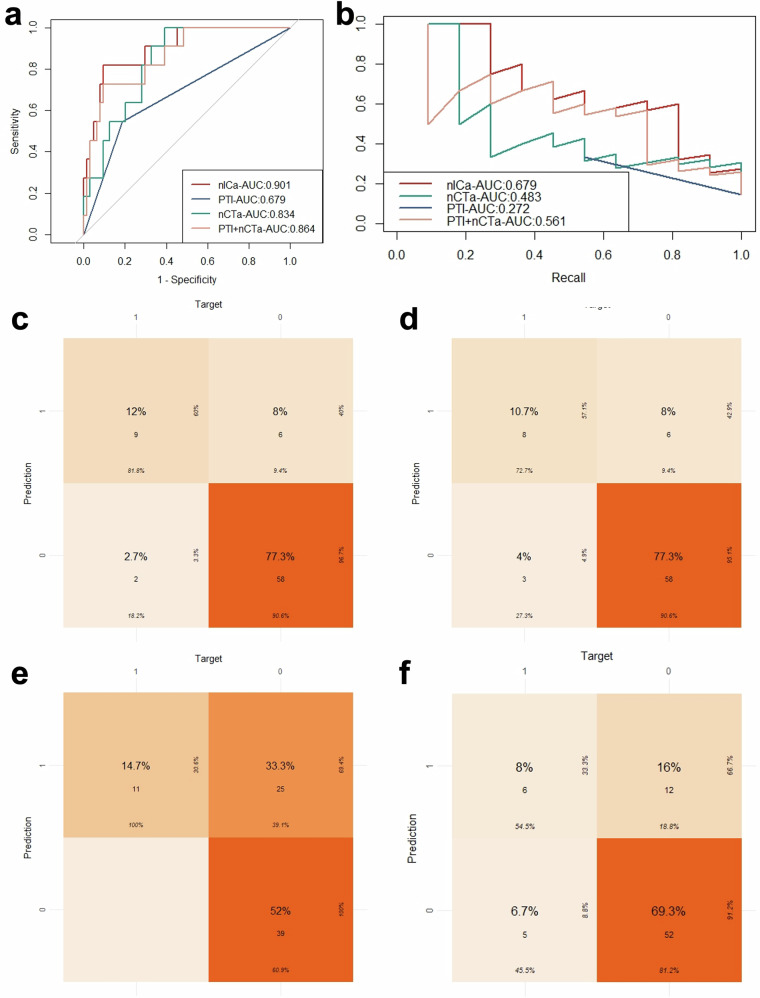
ROC, PR curves, and confusion matrix of four models. **a** Represents ROC curves of nICa, PTI, nCTa, and the combined model (PTI+nCTa), and the AUC was 0.901, 0.679, 0.834, and 0.864, respectively. **b** Represents PR curves of nICa, PTI, nCTa, and the combined model (PTI + nCTa), and the AUC was 0.679, 0.272, 0.483, and 0.561, respectively. **c**–**f** Represent the confusion matrix graphs for four models. **c** shows the nICa model, with a sensitivity of 81.8% (9/11), specificity of 90.6% (58/64), and accuracy of 89.3% (67/75). **d** Shows the combined model (PTI + nCTa), with a sensitivity of 72.7% (8/11), specificity of 90.6% (58/64), and accuracy of 88.0% (66/75). **d** Shows the nCTa model, with a sensitivity of 100% (11/11), specificity of 60.9% (39/64), and accuracy of 66.7% (50/75). **e** Shows the PTI model, with a sensitivity of 54.5% (6/11), specificity of 81.2% (52/64), and accuracy of 77.3% (58/75). ROC, receiver operating characteristic; PR, precision-recall; nCTa, normalised CT attenuation in the pancreatic phase; PTI, peripancreatic tumour infiltration; nICa, normalised iodine concentration in the pancreatic phase

**Fig. 5:**
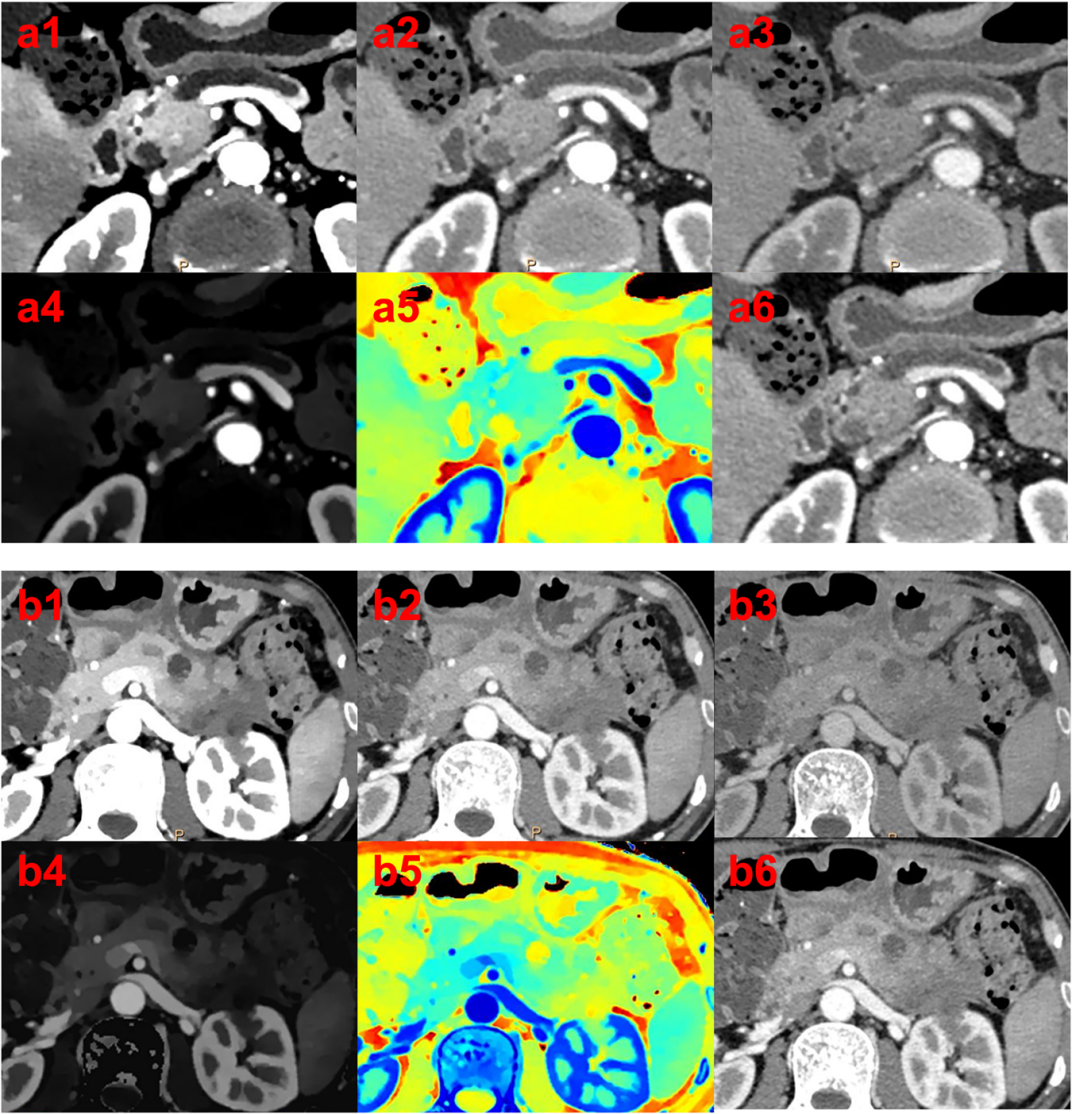
Examples of PDAC patients with high/low TMB. The **a** (1–6), **b** (1–6) Images are VMIs at 40, 70, and 100 keV, IC maps, Zeff images, and conventional CT images. **a**(1–6) A 66-year-old female was diagnosed with PDAC, and the TMB of the tumour was 12.07 mut/Mb. The nICa was 0.137, and the PFS was 14 months. **b** (1–6) A 40-year-old male was diagnosed with PDAC, and the TMB of the tumour was 0.71 mut/Mb. The nICa was 0.007, and the PFS was 3 months. PDAC, pancreatic ductal adenocarcinoma; TMB, tumour mutation burden; VMIs, virtual monoenergetic images; IC, iodine concentration; Zeff, effective atomic number; nICa, normalised iodine concentration in the pancreatic phase; PFS, progression-free survival

ROC curve analysis (Fig. 4a) demonstrated that nICa provided superior predictive performance for TMB, with an area under the ROC curve (AUC-ROC) of 0.901 (cutoff: 0.089), compared with PTI (AUC-ROC = 0.679), nCTa (AUC-ROC = 0.834), and the combined model (PTI + nCTa) (AUC-ROC = 0.864). Confusion matrix analysis (Fig. 4c–f) showed that the nICa model achieved a sensitivity of 81.8%, specificity of 90.6%, accuracy of 89.3%, positive predictive value of 60.0%, and negative predictive value of 96.7%. The NRI and the IDI showed that the nICa model had the best predictive performance compared to the PTI, nCTa, and their combined models (Table 4). The PR curve (Fig. 4b) further supported these findings, with nICa yielding a larger area under the PR curve (AUC-PR = 0.679) and a higher F_1_ score of 0.692 compared with PTI (AUC-PR = 0.272, F_1_ score = 0.414), nCTa (AUC-PR = 0.483, F_1_ score = 0.480), and the combined model (AUC-PR = 0.561, F_1_ score = 0.640). Overall, the nICa model demonstrated the highest efficacy in both ROC and PR curve analyses for predicting TMB levels in patients with PDAC. Figure 5 showed examples of PDAC patients with high/low TMB.

**Table 4:** Performance comparison of predictive models

Model		nICa	PTI	nCTa	PTI + nCTa
Comparison to nICa	NRI	/	0.007^*^	0.002^*^	0.012^*^
	IDI	/	0.005^*^	0.002^*^	0.130
Cutoff value		0.089	/	0.187	/
AUC-ROC (95% CI)		0.901 (0.807–0.995)	0.679 (0.517–0.841)	0.834 (0.731–0.937)	0.864 (0.752–0.975)
AUC-PR (95% CI)		0.679 (0.373–0.882)	0.272 (0.090–0.585)	0.483 (0.223–0.753)	0.561 (0.280–0.808)
F_1_ score		0.692	0.414	0.480	0.640
Accuracy		89.3% (89.1%–89.6%)	77.3% (76.9%–77.8%)	66.7% (66.1%–67.2%)	88.0% (87.7%–88.3%)
Sensitivity		81.8% (59.0%–100%)	54.5% (25.1%–84.0%)	100%(100%–100%)	72.7% (46.4%–99.0%)
Specificity		90.6% (83.5%–97.8%)	81.2% (71.7%–90.8%)	60.9% (49.0%–72.9%)	90.6% (83.5%–97.8%)
Positive predictive value		60.0% (35.2%–84.8%)	33.3% (11.6%–55.1%)	30.6% (15.5%–45.6%)	57.1% (31.2%–83.1%)
Negative predictive value		96.7% (92.1%–100%)	91.2% (83.9%–98.6%)	100% (100%–100%)	95.1% (89.7%–100%)

*nICa* normalised iodine concentration in the pancreatic phase, *nCTa* normalised CT attenuation in the pancreatic phase, *PTI* peripancreatic tumour infiltration, *NRI* net reclassification improvement, *IDI* integrated discrimination improvement, *AUC* area under the curve, *ROC* receiver operating characteristic, *CI* confidence interval, *PR* precision-recall

^*^ The parameter is statistically significant (*p* < 0.05) and is shown in bold type

### Prognostic significance of clinical and radiological characteristics and DLCT parameters

**Fig. 6:**
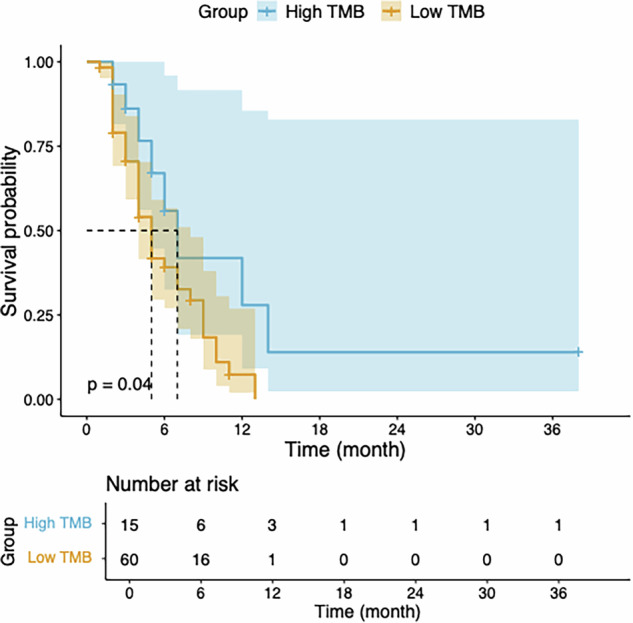
Comparison of PFS predicted by the nICa model. nICa, normalised IC in the pancreatic phase; low TMB, low TMB group predicted by the model; high TMB, high TMB group predicted by the model

The nICa model (Fig. 6) demonstrated significant predictive performance for PFS (*p* = 0.04). According to the nICa model, the high TMB group (15 cases, median PFS: 7 months) exhibited significantly better PFS compared to the low TMB group (60 cases, median PFS: 5 months; *p* = 0.04).

## Discussion

In PDAC, elevated TMB correlated with improved PFS (median PFS: 7 vs 4 months) in patients receiving combination immunotherapy. DLCT–derived nICa emerged as the strongest independent predictor of survival-graded TMB status, achieving superior diagnostic accuracy compared with conventional CT metrics and clinical parameters (AUC: 0.901 vs 0.679–0.864). Notably, nICa-stratified TMB groups mirrored prognostic outcomes, confirming its dual utility as both a predictive biomarker of treatment response and prognostic indicator.

TMB is a well-established independent biomarker for predicting survival in immunotherapy-treated cancers [9–11]. To our knowledge, this is the first study to demonstrate the prognostic significance of TMB in patients with PDAC undergoing combined chemoimmunotherapy. The unique PDAC tumour microenvironment—characterized by dense stroma and dysfunctional vasculature—not only hinders drug penetration but also creates an immune-evasive niche by limiting antigen presentation and suppressing host anti-tumour immunity [25]. Chemotherapy may overcome this barrier by inducing tumour cell death and disrupting immunosuppressive pathways, thereby exposing neoantigens [26]. In patients with high-TMB status, this process likely enhances immunotherapy efficacy, resulting in prolonged PFS [27–29]. These findings support TMB assessment as a potential tool for predicting therapeutic response and guiding personalized treatment strategies in PDAC [30].

In a previous study, there were prognostic differences between the three groups, including high-TMB (≥ 10 mut/Mb, median PFS: 68months), intermediate-TMB (5–10mut/Mb, median PFS: 7 months), and low-TMB (≤ 5mut/Mb, median PFS: 8 months) cohorts [31], proving the prognostic value of TMB in PDAC. However, there was a discernible difference in cohort composition between this previous work and our own: while all patients in the previous study underwent surgical resection, the vast majority in our study presented with locally advanced or metastatic, unresectable PDAC. This critical distinction likely influenced the observed TMB distribution difference and may, in part, explain the divergence in TMB stratification thresholds between the two studies. Though the FDA approval of pembrolizumab for adult and paediatric patients with TMB ≥ 10 mut/Mb [32], it remains unclear whether separate thresholds should be adopted for specific tumour types or whether a universal threshold (such as 10 mut/Mb) should be applied to all tumours [7]. Given the low rate in PDAC with high TMB (≥ 10 mut/Mb) [31, 33], there is an urgent clinical need to adjust to a more appropriate threshold. Notably, whereas the thresholds in the previous study [31] were defined a priori, our approach utilized cutoff points informed by survival analyses. By establishing the optimal TMB cutoff through PFS analysis, it might be a more objective classification tool and a prognostic indicator specific to more advanced PDAC. Future studies with larger, multi-stage PDAC cohorts will be required to fully elucidate the prognostic significance of TMB.

DLCT demonstrates superior material discrimination capabilities compared to conventional CT, particularly in iodine quantification, due to its advanced multi-parametric quantitative analysis [34]. Our findings revealed that all tumour enhancement parameters, whether derived from conventional CT or DLCT during pancreatic phase imaging, effectively predict TMB levels in PDAC. Confirming Hu et al’s observations in acute pancreatitis [35], pancreatic-phase parameters more precisely characterize pancreatic microcirculation than portal venous-phase measurements. This enhanced accuracy likely stems from the parameters’ predominant reflection of arterial flow and tumour vascular morphology, both of which show strong correlation with microvascular density [36]. The nICa serves as a direct indicator of tumour vascularity and perfusion, as iodine constitutes the principal component of contrast agents [37, 38]. Zhang et al’s reported positive association between VEGFA expression and TMB [39], further supporting nICa emerged as the most reliable microvascular density surrogate and TMB predictor observed in our study. Despite this study demonstrating that elevated TMB correlates with poorer overall survival, which might appear contradictory to our findings (elevated TMB correlated with favourable PFS), this discrepancy arises from critical therapeutic differences between cohorts. Notably, the comparator study lacked immunotherapy exposure, whereas our cohort predominantly received combination immunotherapy. Recent pan-cancer analyses have corroborated that high TMB portends a favourable outcome exclusively in patients with immunotherapy, whereas in patients without immunotherapy, higher TMB may be linked to poorer survival [40]. This phenomenon underscores the necessity of interpreting TMB data considering immunotherapeutic intervention, and further highlights the clinical significance of accurate, noninvasive TMB prediction in guiding optimized treatment choices for PDAC. Notably, parameters at the portal venous phase demonstrated systematically higher values than pancreatic phase measurements across all cohorts, mirroring Liu et al’s observations of delayed peak enhancement in PDAC [14]. This phenomenon likely stems from contrast retention in fibrotic tissues, explaining the temporal shift in enhancement kinetics [41]. Importantly, the high-TMB group exhibited consistently lower normalised parameters than the low-TMB group within each phase, suggesting reduced fibrosis burden—a finding that may account for their superior PFS and corroborates Shi et al’s fibrosis-prognosis association [42].

We further identified an inverse relationship between PTI and TMB. Çorbacı et al [43] demonstrated significantly elevated CD8^+^ T cell infiltration in PTI-positive patients, while Zhang et al reported similar immune patterns in low-TMB cases [39]. These collective findings support a potential mechanism wherein PTI facilitates immune cell trafficking through the fibrotic stroma, increasing tumour antigen exposure and augmenting antitumour immune responses.

Several limitations of this study should be acknowledged. First, the retrospective single-centre design with a limited cohort size introduces possible selection bias. Second, tumour spatial heterogeneity may have caused discrepancies between imaging measurements and pathological sampling regions. Third, our exclusive use of single-vendor DLCT equipment may affect the generalizability. These constraints highlight the need for future large-scale, multicenter prospective validation studies. Additionally, the sample size precluded meaningful subgroup analysis of patients receiving combination immunotherapy.

In conclusion, our findings established elevated TMB as a positive prognostic biomarker for PFS in PDAC. The DLCT-derived nICa parameter emerges as a non-invasive predictor of survival-graded TMB status and clinical outcomes, demonstrating superior predictive performance to conventional CT and clinical models. This imaging biomarker holds significant potential to guide therapeutic decision-making and improve prognostic assessment in PDAC management.

## Data Availability

The original data generated in this study can be requested from the corresponding author. For any researchers interested in obtaining these data, we encourage you to contact the corresponding author via email at fengsht@mail.sysu.edu.cn to discuss the specific conditions for data access and possible measures for privacy protection.

## Abbreviations

AUC: Area under the curve
BMI: Body mass index
CA 19-9: Carbohydrate antigen 19-9
CI: Confidence interval
DLCT: Dual-layer spectral detector CT
IC: Iodine concentration
ICCs: Intraclass correlation coefficients
IDI: Integrated discrimination improvement
LASSO: Least absolute shrinkage and selection operator
NRI: Net reclassification improvement
OR: Odds ratio
PDAC: Pancreatic ductal adenocarcinoma
PFS: Progression-free survival
PR: Precision-recall
ROC: Receiver operating characteristic
TMB: Tumour mutation burden
VMIs: Virtual monoenergetic images
Zeff: Effective atomic number
